# Challenging Aspects of Familial Adenomatous Polyposis With Malignant Transformation: A Report of Two Cases

**DOI:** 10.7759/cureus.98789

**Published:** 2025-12-09

**Authors:** Fatima Zahra Belabbes, Sara Mounsif, Rania Arja, Basma Elkhannoussi, Imane Ben Elbarhdadi

**Affiliations:** 1 Department of Gastroenterology and Hepatology, Mohammed VI University of Health Sciences, Cheikh Khalifa International University Hospital, Casablanca, MAR; 2 Research Unit, Mohammed VI Center for Research and Innovation, Rabat, MAR; 3 Department of Pathology, Mohammed VI University of Health Sciences, Cheikh Khalifa International University Hospital, Casablanca, MAR

**Keywords:** bowel cancer screening, colorectal cancer, lower endoscopy, polyps, familial adenomatous polyposis

## Abstract

Familial adenomatous polyposis (FAP) is a genetic disorder characterized by the early onset of hundreds of polyps in the gastrointestinal tract, mainly in the colon and rectum. Despite its rarity, data on FAP in Morocco remain limited due to the absence of a national registry and comparative case series.

We present two cases of FAP diagnosed in Morocco. The two patients, one male and one female, had a mean age of 34 years. Initial colonoscopy revealed more than 100 polyps for both patients. The polyps varied in size and morphology, including flat, sessile, and pedunculated forms, and were distributed throughout the colon and rectum, and all showed adenomatous features on virtual chromoendoscopy. Histopathological examination of the polyps revealed a range of findings, from low-grade tubulovillous adenomas to moderately differentiated rectal adenocarcinoma. Esophagogastroduodenoscopy (EGD) revealed duodenal polyps, with histology confirming tubular adenomas exhibiting both low- and high-grade dysplasia.

Both patients underwent surgery: one had a subtotal colectomy with ileorectal anastomosis, and the other a total proctocolectomy with ileoanal anastomosis.

The clinical evolution was marked by the development of colon adenocarcinoma in one patient and rectal adenocarcinoma in the other. Regular surveillance of the duodenal polyps was recommended every 1-2 years.

## Introduction

Familial adenomatous polyposis (FAP) is a rare autosomal dominant genetic disorder characterized by the early onset of hundreds of polyps in the gastrointestinal tract, primarily in the colon and rectum, starting at an early age.

Although FAP represents only about 1% of colorectal cancers, it carries a high risk of early-onset malignancy without proper management [1]. Proper management includes regular endoscopic surveillance, timely surgical intervention (sometimes including prophylactic colectomy), follow-up for extra-colonic manifestations, and genetic counseling for patients and at-risk family members.

Caused by APC gene mutations that disrupt cell proliferation, FAP underscores the need for genetic testing. Yet, in 20% of cases, the causative mutation remains unidentified, complicating management [2].

In Morocco, where genetic diversity may affect disease presentation, careful diagnosis and targeted screening are crucial for at-risk individuals. Although FAP is uncommon, it is particularly challenging, especially in some countries, due to delayed diagnosis, limited access to genetic testing, the absence of a national colorectal cancer screening program, and patients often being lost to follow-up.

We present two cases of FAP diagnosed and managed in this context, highlighting the diagnostic and therapeutic challenges, as well as the importance of early recognition and family-based management.

## Case presentation

Case 1

A 46-year-old woman presented to our gastroenterology clinic with a five-year history of rectal bleeding and chronic abdominal pain.

Her medical history revealed that 15 years prior, at the age of 31, she was evaluated for intermittent isolated rectal bleeding. Colonoscopy at that time revealed more than 100 adenomatous polyps distributed throughout the colon and rectum, suggesting the diagnosis of FAP. Histopathological analysis confirmed the adenomatous nature of the polyps, including one colonic polyp with malignant transformation into adenocarcinoma, staged as pT2N0M0.

The patient underwent a subtotal colectomy with ileorectal anastomosis; however, surgical records were unavailable. No upper gastrointestinal endoscopy was performed, genetic testing was declined, and no postoperative surveillance was conducted as the patient was lost to follow-up and refused further care.

On admission, the patient was in good general condition, with a performance status of 0 and normal vital signs. Digestive examination was unremarkable, with a soft, non-tender abdomen and normal digital rectal findings. Laboratory tests, including hemoglobin, were within normal limits.

**Figure 1:**
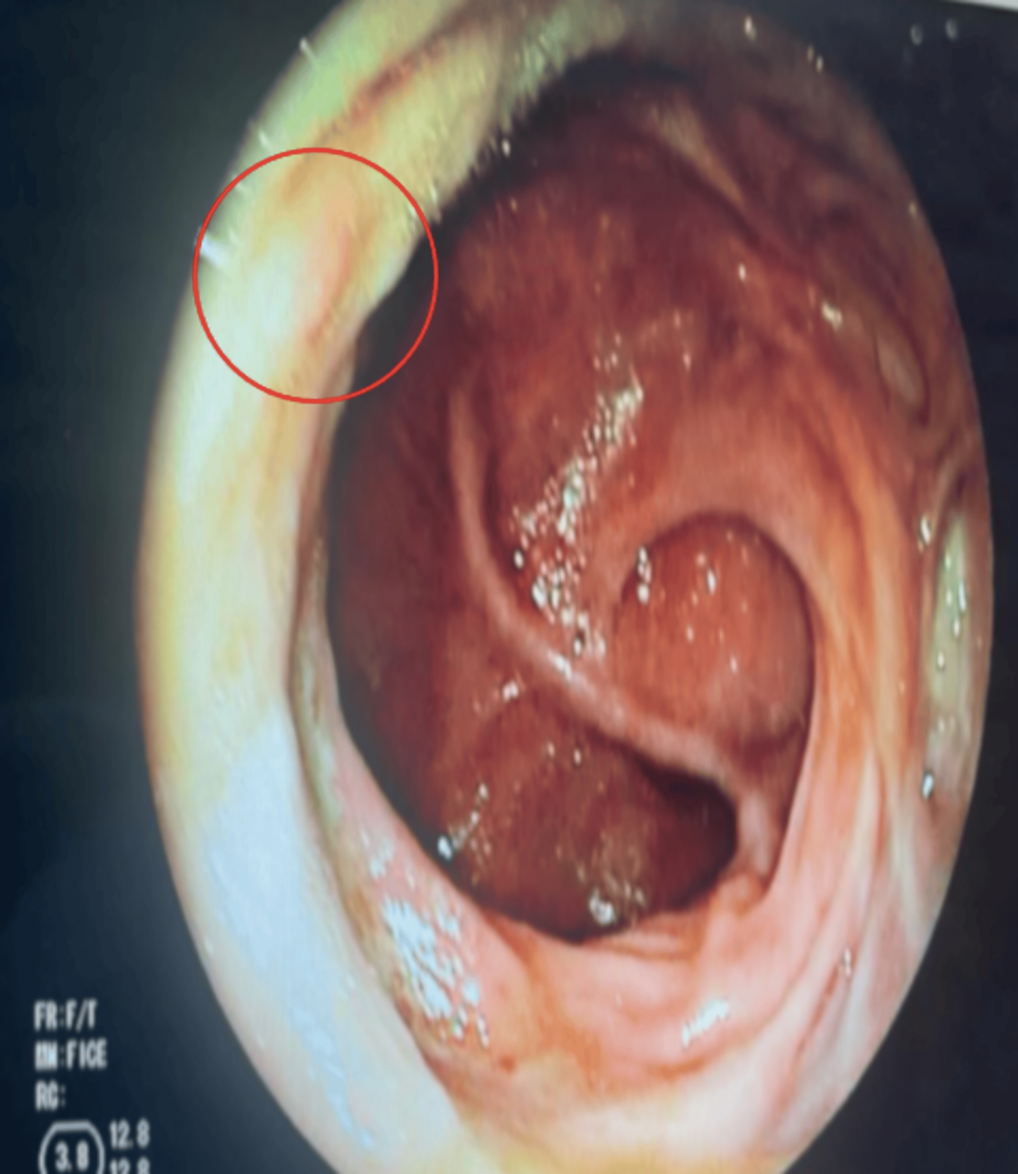
Colonoscopy image showing an 8 mm polyp in the lower rectum, classified as Is by the Paris classification and IIA by the Connect classification using virtual chromoendoscopy

**Figure 2:**
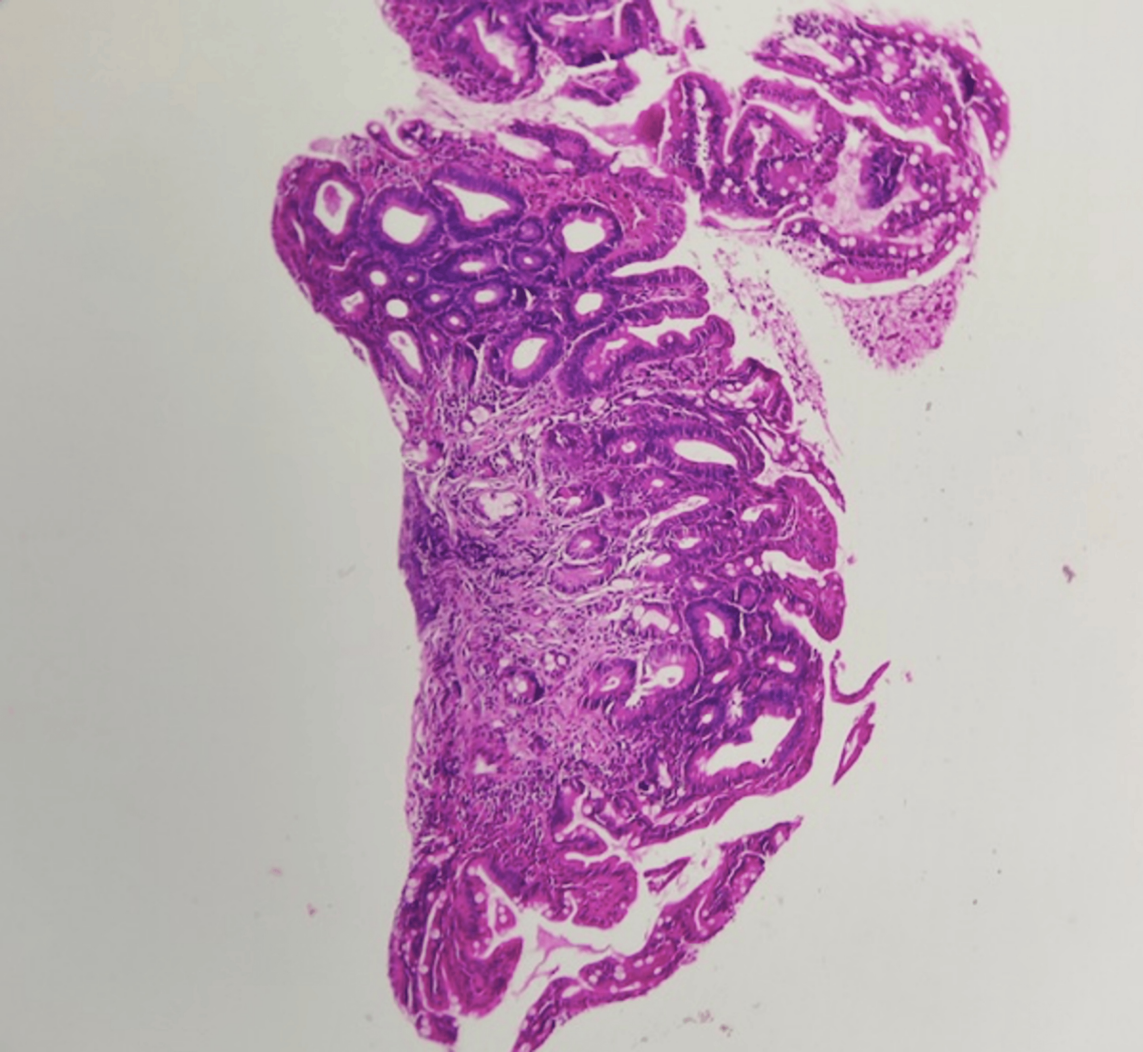
Histopathological analysis of the resected rectal polyp revealing a low-grade dysplastic adenoma

Rectosigmoidoscopy was performed and revealed an 8 mm sessile polyp in the lower rectum (Figure 1), which was resected endoscopically. Histopathological examination confirmed a tubular adenoma with low-grade dysplasia, without evidence of invasion (Figure 2).

**Figure 3:**
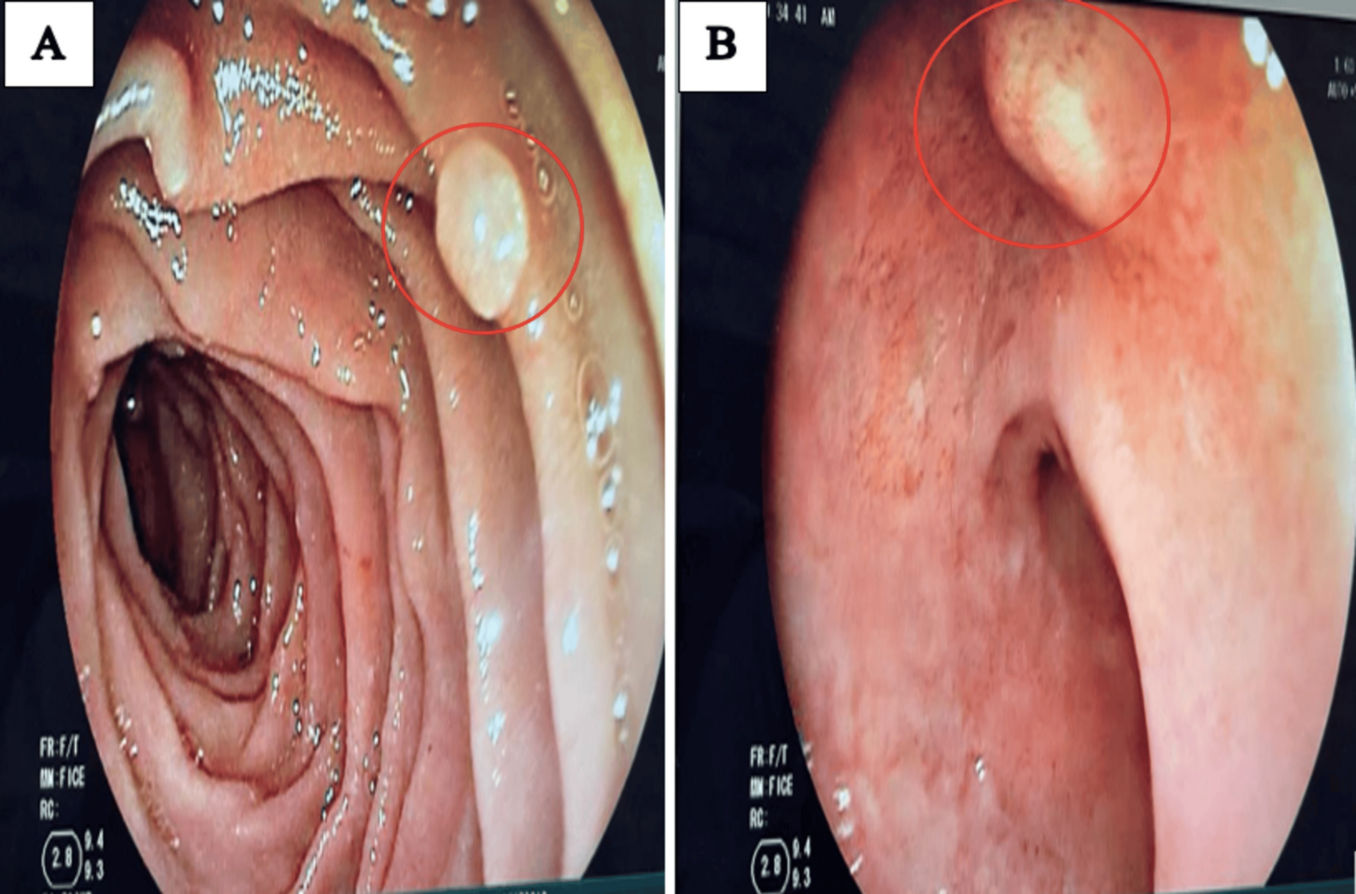
Endoscopic image showing millimetric duodenal polyps in the second portion of the duodenum (D2) (A) and the duodenal bulb (B), with adenomatous appearance on chromoendoscopy, classified as Is according to the Paris classification

As part of the FAP assessment, an esophagogastroduodenoscopy (EGD) revealed multiple diminutive duodenal polyps (Figure 3). Histopathological examination confirmed tubulovillous adenomas with high-grade dysplasia.

Screening for extraintestinal manifestations of FAP included dermatological, ophthalmological, and dental evaluations, all of which revealed no abnormalities.

Genetic testing for the patient and her family has been recommended but not yet performed. Colonoscopy of the patient's mother (55 years old) and sister (22 years old) revealed no polyps.

The patient is currently managed with regular clinical and endoscopic surveillance of both the lower and upper gastrointestinal tract.

Case 2

The second patient is a 22-year-old man who was referred to our clinic for the evaluation of symptomatic iron deficiency anemia. He has no significant past medical history and a family history of endometrial cancer in his grandmother.

Clinically, he reported symptoms consistent with anemia, including exertional dyspnea, palpitations, and fatigue for the past nine years, with no overt bleeding or digestive symptoms. General examination on admission showed a good overall condition, mild skin and mucosal pallor, and normal vital signs. Clinical and abdominal examinations were unremarkable. No active bleeding or palpable masses were found on digital rectal examination.

Laboratory tests revealed microcytic anemia with a hemoglobin level of 10 g/dL, a mean corpuscular volume (MCV) of 73.1 fL, and a normal platelet count of 335,000/mm³.

An endoscopic evaluation was performed. On EGD, more than 20 polyps of millimetric size were observed in the duodenum. Biopsy of the polyps confirmed a tubular adenoma with low-grade dysplasia.

**Figure 4:**
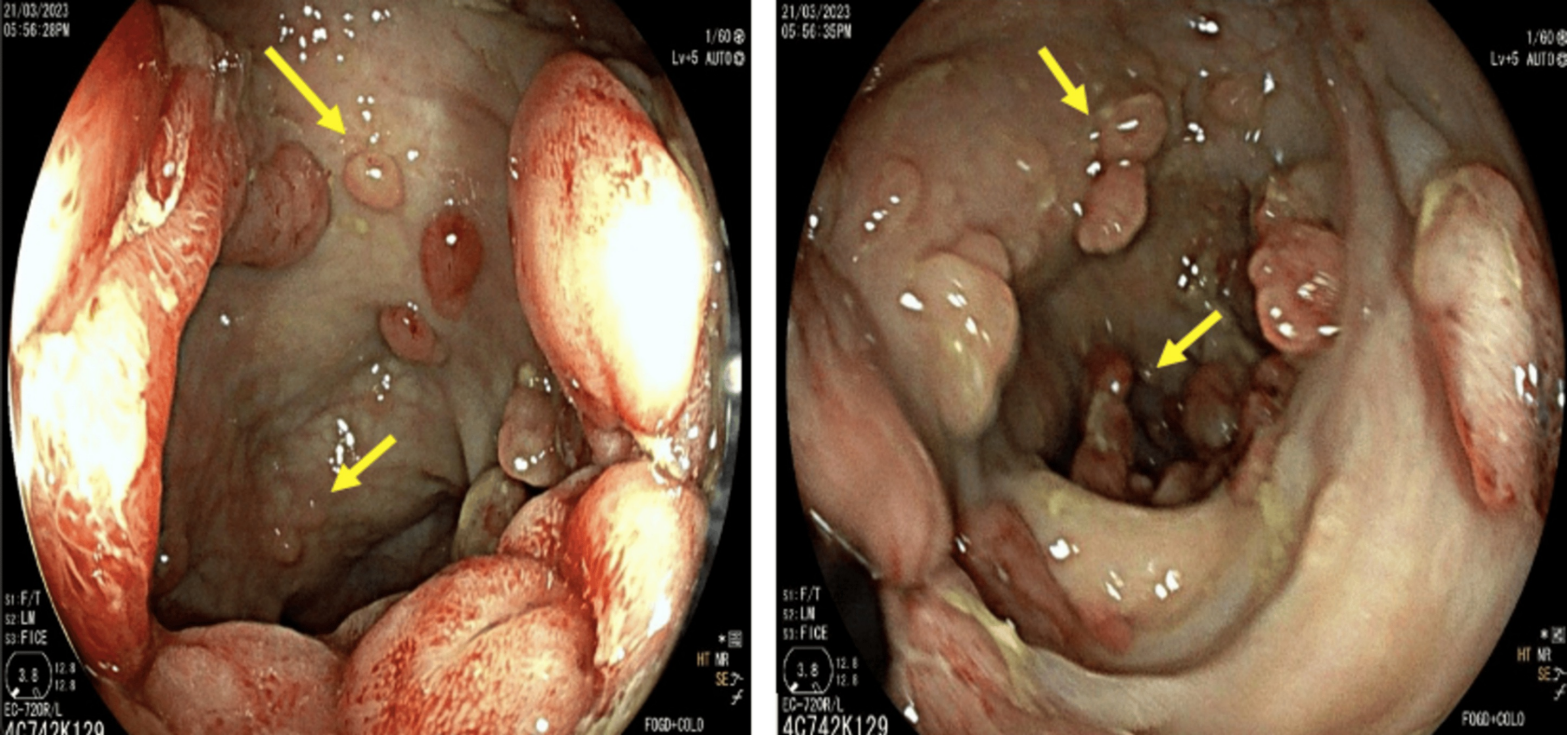
Colonoscopy image showing multiple sessile and pedunculated polyps, both centimetric and millimetric, classified as IIA by the Connect classification

**Figure 5:**
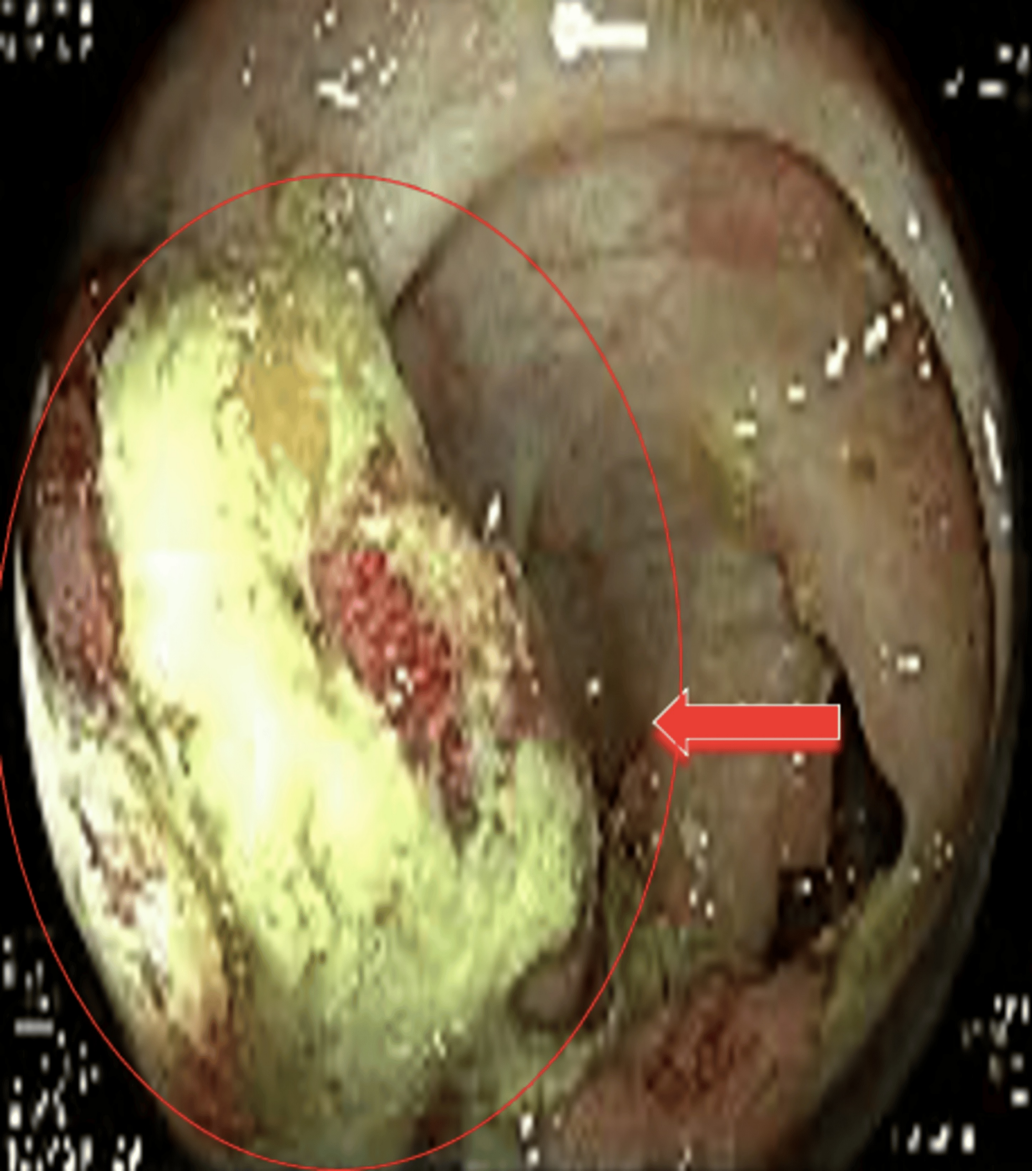
Colonoscopy image showing an exophytic tumor process in the upper rectum, located 10-11 cm from the anal margin

Colonoscopy revealed extensive adenomatous polyposis involving the entire recto-colic mucosa, with over 100 adenomatous-appearing polyps observed on virtual chromoendoscopy using blue laser imaging (BLI) (Figure 4), associated with a friable, exophytic, and ulcerated lesion in the rectum (Figure 5).

**Figure 6:**
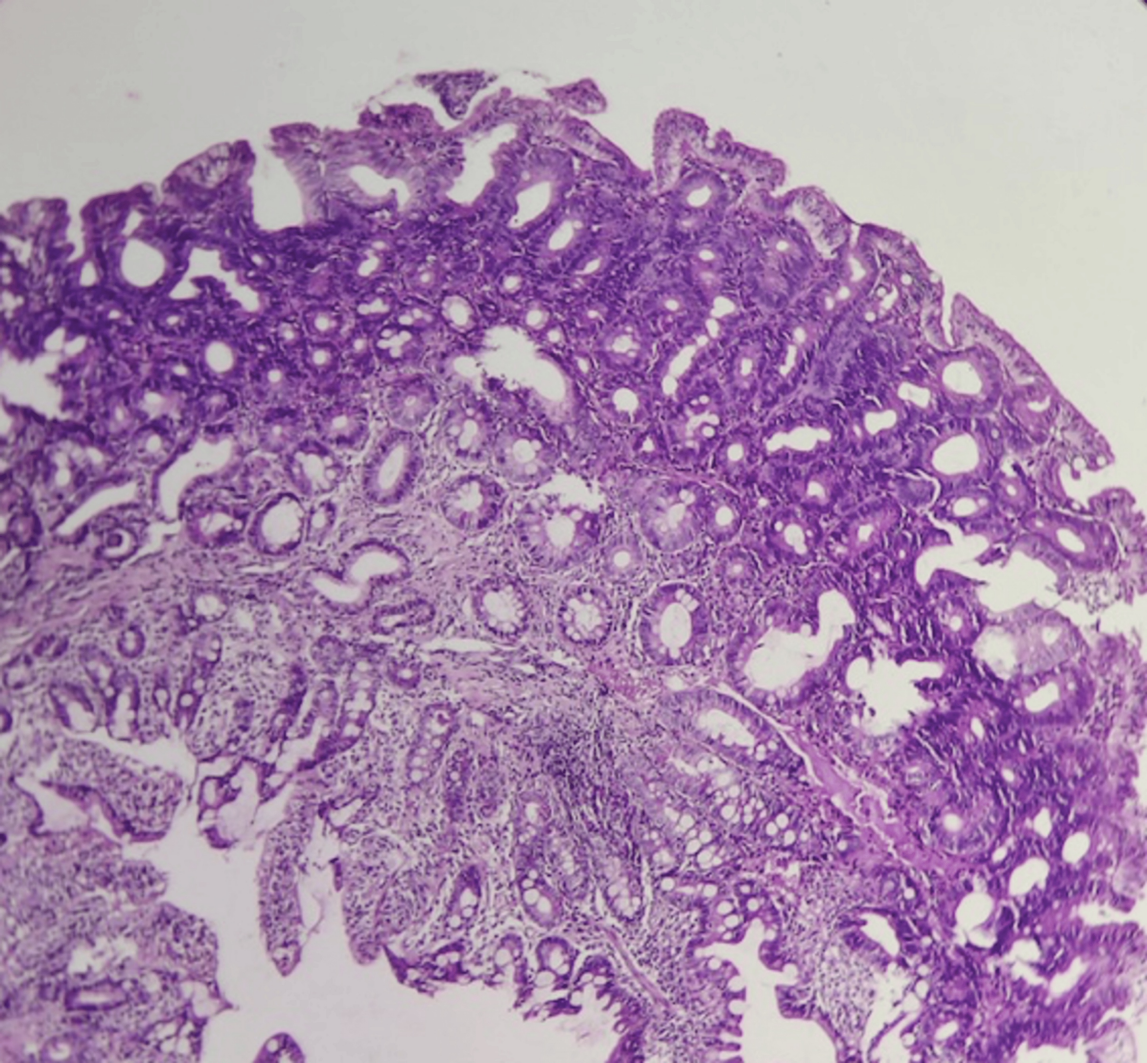
Histological appearance of a high-grade dysplastic adenoma

Histopathological examination of the polyps revealed adenomas with low- and high-grade dysplasia (Figure 6), and histopathological analysis of the rectal lesion confirmed the presence of a moderately differentiated rectal adenocarcinoma.

**Figure 7:**
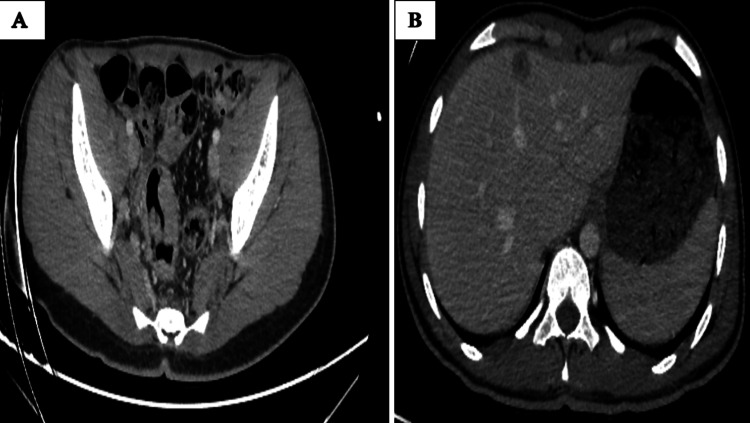
Axial contrast-enhanced CT images of the patient showing the circumferential thickening of the rectosigmoid junction (A) with two hypodense target lesions in hepatic segments VI (12 mm) and IV (15 mm), demonstrating mild enhancement after contrast injection (B)

The staging workup, including thoraco-abdomino-pelvic CT scan, revealed thickening of the rectosigmoid junction with secondary-appearing hepatic lesions in segments IV and VI (Figure 7).

The diagnosis of FAP with malignant transformation was established. Screening for other extraintestinal manifestations, including dermatological, ophthalmological, and dental evaluations, showed no abnormalities. Genetic testing was requested but not performed.

Following discussion at the digestive oncology multidisciplinary team meeting, the patient underwent total proctocolectomy with ileoanal anastomosis and hepatic metastasectomy. The postoperative course was uneventful.

Histopathological examination of the surgical specimen revealed a well-differentiated, low-grade adenocarcinoma infiltrating the rectal wall up to the perirectal adipose tissue, staged as pT3N1bM1a according to the TNM classification. Chemotherapy based on FOLFOX (folinic acid, 5-fluorouracil, and oxaliplatin) was subsequently initiated.

The patient is currently being followed in outpatient consultation, with no reported signs of complications.

## Discussion

FAP is a disorder characterized by the development of multiple adenomas, typically from hundreds to thousands, predominantly in the colon and rectum [3].

Classic FAP has an estimated incidence of one in 7,000 to one in 30,000 live births, affecting men and women equally [4]. It typically manifests in young adults, sometimes as early as childhood, with adenomas often developing by adolescence [5-7].

The main risk in FAP is the progression of these adenomas to colorectal cancer, which often arises by the fourth decade, much earlier than in sporadic cases, with 70-80% of tumors located in the left colon [8].

FAP is an autosomal dominant disorder caused by mutations in the APC tumor suppressor gene. APC is a key regulator of the Wnt signalling pathway, controlling β-catenin-dependent gene expression and consequently governing cell proliferation and differentiation. These mutations disrupt normal growth regulation, leading to adenoma formation [9].

Around 25% of FAP cases result from de novo APC mutations, occurring in individuals with no previous family history or clinical signs of the disorder [9]. This can complicate and delay diagnosis in patients who initially appear not to be at risk.

Symptoms of FAP are nonspecific and may include constipation or diarrhea, lower gastrointestinal bleeding, mucous discharge, abdominal pain, bloating, and weight loss [10,11].

FAP is usually suspected during a colonoscopy, often performed in case of symptoms or a family history of polyps or colorectal cancer. The colon and rectum usually reveal numerous adenomatous polyps, typically numbering 100 or more.

A milder form, known as attenuated familial adenomatous polyposis (AFAP), is characterized by fewer than 100 polyps, slower polyp progression, and a later onset of cancer [9].

Historically, FAP was diagnosed clinically, but recent studies have demonstrated the clinical utility of multi-gene panel testing (MGPT). Genetic testing offers several advantages, including the definitive confirmation of FAP, exclusion of other polyposis syndromes, identification of at-risk relatives, and more accurate cancer risk assessment based on specific APC variants [12].

Patients with FAP have an increased risk of developing various intestinal and extraintestinal manifestations, including gastroduodenal polyps, desmoid tumors, congenital hypertrophy of the retinal pigment epithelium (CHRPE), fibromas, lipomas, epidermoid cysts, nasal angiofibromas, thyroid carcinomas, hepatoblastomas, pancreatobiliary tumors, and brain tumors [13]. Due to the heterogeneity of extraintestinal manifestations, systematic evaluation is crucial in all FAP patients.

Upper gastrointestinal endoscopy should also be performed to screen for upper digestive polyps [12]. Duodenal and periampullary polyps have a high risk of adenocarcinoma, while gastric fundic polyps rarely become malignant (<1%) [14,15].

Therapeutic management of FAP depends on the extent of polyposis and the presence of colorectal cancer at diagnosis.

Guidelines for the management of FAP vary among professional societies, highlighting the challenge of balancing surgical morbidity, quality of life, and cancer risk.

Absolute indications include cancer or severe polyposis, while relative ones concern moderate or progressive disease. The choice between ileorectal anastomosis and restorative proctocolectomy with ileal pouch-anal anastomosis (IPAA) is guided mainly by the extent of rectal polyposis [12].

Surveillance in FAP is tailored to phenotype and surgical status, with intervals adjusted to polyp burden. Most guidelines recommend colonoscopy every 1-2 years or 1-3 years, starting at ages of 10-12 in classic FAP, with upper gastrointestinal surveillance from 20 to 25 years [12]. Post-colectomy, follow-up of the rectum or pouch is mandatory [12].

In Morocco, the absence of a national colorectal cancer screening program may partly account for delayed diagnoses and advanced disease at presentation. In addition, access to genetic testing can be limited and challenging, which may complicate the management of FAP. Through these case reports, we aim to highlight the importance of enhanced surveillance and targeted screening in at-risk individuals.

## Conclusions

FAP is a hereditary condition with numerous adenomas and high early colorectal cancer risk. Management relies on genetic testing, regular surveillance, and timely surgery. Given the lack of national colorectal screening programs in some regions, delayed diagnosis remains a challenge. These case reports underscore the importance of vigilant surveillance and targeted screening in at-risk individuals.
